# The Physicians’ Leisure Library Series

**Published:** 1886-10

**Authors:** 


THE PHYSICIANS' LEISURE LIBRARY SERIES. Published by Geo. S. Davis, Detroit, Michigan.

This series, when completed, will consist of twelve small, handy pamphlets on special subjects. Six of the set are now offered to the profession.

INHALERS, INHALATIONS AND INHALANTS. By Beverly Robinson, M. D. The Use of Electricity in the Removal of Superfluous Hair and the Treatment of Various Facial Blemishes. By Geo. Henry Fox, M. D. New Medications. By Dujardin Beaumetz, M. D. Translated by E. P. Hind, M. D. The Modern Treatment of Ear Diseases. By Samuel Sexton, M. D. Spinal Irritation. By William A. Hammond, M. D. The Modern Treatment of Eczema. By Henry S. Pifford, M. D.

Each little volume contains, in clear and concise form, the latest knowledge upon the subject it represents, and as the subjects are handled by men who are acknowledged authorities in their special branches, the information given is at once reliable and worthy of study. Most of the little works can be read at one sitting, and their cheapness, together with the names of the authors, must recommend them to the favorable notice of every intelligent practitioner. The series deserve a ready sale.

